# Editorial: Harm and Benefit of Plant and Fungal Secondary Metabolites in Food Animal Production

**DOI:** 10.3389/fvets.2018.00036

**Published:** 2018-03-05

**Authors:** Michael D. Flythe

**Affiliations:** ^1^Forage-Animal Production Research Unit, Agricultural Research Service, United State Department of Agriculture, Lexington, KY, United States; ^2^Department of Animal and Food Sciences, University of Kentucky, Lexington, KY, United States

**Keywords:** plant secondary metabolites, food, agriculture, natural products, plant toxins, animal nutrition, ergotism, essential oils

**Editorial on the Research Topic**

**Harm and Benefit of Plant and Fungal Secondary Metabolites in Food Animal Production**

The diets of agricultural animals provide energy and components for anabolism, as well as vitamins and minerals. Other impacts of the diet are caused by plant and fungal secondary metabolites. Toxins have always been a concern in animal agriculture. Ergot alkaloids are central to five articles in this *Research Topic* (Jackson et al.; Jia et al.; Shappell et al.; Coufal-Majewski et al.; Aiken et al.). Coufal-Majewski et al. state that as much as 20% of the wheat produced in Canada is infected with ergot-producing *Claviceps* species. The effects of ergot alkaloids on livestock include tremors, vasoconstriction, and gangrene, and less obvious impacts on growth and reproduction. The review covers impacts on livestock as well as the general information on *Claviceps* fungi. Importantly, the analytical methods we need to detect and quantify ergot alkaloids in feeds are also described (Coufal-Majewski et al.).

Ergot alkaloids are also the toxins involved in fescue toxicosis, which impacts forage-based livestock production in the USA, Australia, and New Zealand (Coufal-Majewski et al.). The tall fescue endophyte, *Epichloë coenophiala*, produces ergot alkaloids, which cause the toxicosis in grazing animals. The primary symptom of fescue toxicosis is vasoconstriction, which causes heat stress and gangrenous lameness. Jackson et al. conducted a tall fescue grazing experiment with steers and examined blood parameters. Depressed prolactin is diagnostic of fescue toxicosis, but they found that albumin, cholesterol, and red blood cell number were also altered throughout the trial, while other parameters changed transiently or only after prolonged exposure. Many of the negative effects of fescue toxicosis are associated with poor blood flow from vasoconstriction. Two of the contributions examined vasoconstriction (Jia et al.; Aiken et al.). Both dealt with the interaction of ergot alkaloids and another category of secondary metabolites, isoflavones from red clover. Aiken et al. examined the effects of ergot alkaloids and clover isoflavones on two arteries in goats *in vivo*. The other study by Jia et al. employed ergot alkaloids and specific isoflavones in an *in vitro* assay with the mesenteric artery and vein. In the *in vivo* study (Aiken et al.), the isoflavones reversed vasoconstriction caused by the ergot alkaloids, but in the *in vitro* (Jia et al.) study, they did not. The differences between the experiments are many, but notably include peripheral versus visceral vasculature and the metabolism of the compounds that occurs *in vivo*. If isoflavones do, in fact, reverse vasoconstriction in fescue toxicosis, it would explain the long-standing observation that cattle performance on tall fescue improves when there is clover in the pasture.

The investigations into clover isoflavones by our research group were originally initiated to explore isoflavones as an alternative to feed antibiotic growth promoters. The antimicrobial action of the isoflavones in these forage plants is similar to those of the secondary metabolites of a horticultural crop, the hops plant. The *Research Topic* includes a review of hops bitter acids as antimicrobial ruminant feed additives (Flythe et al.). The bitter acids are potent antimicrobials, and, depending on the diet, the benefits include decreased rumen ammonia, acidity, methane production, and improved average daily gain.

There is a great need for antimicrobials in modern animal agriculture, and many other non-forage plants also contain antimicrobial compounds that are beneficial in terms of animal growth, waste product reduction, or the suppression of pathogens. It is common for compounds to be extracted to produce essential oils, which are the subject of review by O’Bryan et al. Animal industries are challenged with ensuring the safety of their food products, and antimicrobials play a role in food safety strategies. The plethora of plant essential oils offers alternatives to clinically important antibiotics. However, the mechanisms of action must be understood to maximize the utility of compounds. O’Bryan et al. describe common physiological targets of essential oils, such as the cell envelope and the respiratory chain, in important food-borne pathogens.

We must be careful not to categorize a plant as strictly beneficial or harmful. Red clover offers a potential mitigation strategy for the harmful effects of tall fescue alkaloids, but red clover can also contain harmful alkaloids (Kagan). Kagan describes the historical and current work on *Rhizoctonia leguminicola*, a fungal pathogen that causes blackpatch in red clover. Infected plants can contain the alkaloids slaframine, which causes profuse salivation, and swainsonine, which causes neurological problems. The review offers several risk-reduction strategies for blackpatch and slobbers, but little is known about *R. leguminicola* and the associated toxicosis (Kagan).

Furthermore, we must be careful not to categorize even a particular plant or a fungal compound as strictly beneficial or harmful. Some fungal and plant secondary metabolites have multiple biological effects. Isoflavones, for example, are antimicrobial, antioxidant, and improve blood flow, but they are also estrogenic (Shappell et al.). The estrogenic activity is beneficial to ruminant growth and blood flow. The isoflavones in soy hulls fed to steers led to an increased estrogenic activity in the blood and improved performance beyond estradiol-implanted steers (Shappell et al.). However, decreased reproductive success has long been noted in animals on high-isoflavone diets. Thus, different recommendations regarding isoflavones might be made for stocker calves versus cow herds.

One of the most well-known harmful plant metabolites is nitrate. Forages can accumulate nitrate, which is reduced to nitrite by rumen bacteria (Anderson et al.). The nitrite is absorbed by the host and binds hemoglobin to form methemoglobin and decreases its ability to carry oxygen in the blood. Anderson et al. point out that even ingested nitrate might have desirable outcomes. It was previously determined that exogenous nitrate or nitro-compounds could reduce rumen methane, a major greenhouse gas. Anderson et al. showed that forages that naturally contained nitrate or nitro-compounds reduced methane production by rumen microorganisms by as much as 87%. It is unclear if nitrate and nitro-rich forages can be applied as a methane mitigation technology without risk to the animals.

It has long been clear that the impacts of plants on food animals go beyond nutrition *sensu stricto*. The harm or benefit of a plant or a fungal secondary metabolite depends on many factors.

## Author Contributions

The author is solely responsible for the content of the editorial.

## Conflict of Interest Statement

The author declares that the research was conducted in the absence of any commercial or financial relationships that could be construed as a potential conflict of interest.

